# Imaging delays among medical inpatients in Toronto, Ontario: A cohort study

**DOI:** 10.1371/journal.pone.0281327

**Published:** 2023-02-03

**Authors:** Emily Bartsch, Saeha Shin, Surain Roberts, Thomas E. MacMillan, Michael Fralick, Jessica J. Liu, Terence Tang, Janice L. Kwan, Adina Weinerman, Amol A. Verma, Fahad Razak, Lauren Lapointe-Shaw

**Affiliations:** 1 Department of Medicine, University of Toronto, Toronto, Ontario, Canada; 2 Unity Health Toronto, Toronto, Ontario, Canada; 3 Division of General Internal Medicine, Toronto Western Hospital, University Health Network, Toronto, Ontario, Canada; 4 Department of Medicine, Sinai Health System, Toronto, Ontario, Canada; 5 Division of General Internal Medicine, Toronto General Hospital, University Health Network, Toronto, Ontario, Canada; 6 Institute for Better Health, Trillium Health Partners, Mississauga, Ontario, Canada; 7 Department of Medicine, Sunnybrook Health Sciences Centre, Toronto, Ontario, Canada; 8 Division of General Internal Medicine, St. Michael’s Hospital, Unity Health Toronto, Toronto, Ontario, Canada; 9 Institute of Health Policy, Management, and Evaluation, University of Toronto, Toronto, Ontario, Canada; Universitatsklinikum Schleswig Holstein Campus Lubeck, GERMANY

## Abstract

**Background:**

Imaging procedures are commonly performed on hospitalized patients and waiting for these could increase length-of-stay. The study objective was to quantify delays for imaging procedures in General Internal Medicine and identify contributing patient, physician, and system factors.

**Methods:**

This was a retrospective cohort study of medical inpatients admitted to 5 hospitals in Toronto, Ontario (2010–2019), with at least one imaging procedure (CT, MRI, ultrasound, or peripherally-inserted central catheter [PICC] insertion). The primary outcome was time-to-test, and the secondary outcome was acute length-of-stay after test ordering.

**Results:**

The study cohort included 73,107 hospitalizations. Time-to-test was longest for MRI (median 22 hours) and shortest for CT (median 7 hours). The greatest contributors to time-to-test were system factors such as hospital site (up to 22 additional hours), location of test ordering (up to 10 additional hours), the timing of test ordering relative to admission (up to 13 additional hours), and ordering during weekends (up to 21 additional hours). Older patient age, having more comorbidities, and residence in a low-income neighborhood were also associated with testing delays. Each additional hour spent waiting for a test was associated with increased acute length-of-stay after test ordering, ranging from 0.4 additional hours for CT to 1.2 hours for MRI.

**Conclusions:**

The greatest contributors to testing delays relate to when and where a test was ordered. Wait times affect length-of-stay and the quality of patient care. Hospitals can apply our novel approach to explore opportunities to decrease testing delays locally.

## Background

Medical imaging plays a critical role in the diagnosis and management of patients admitted to hospital. Nearly two-thirds of patients admitted to general medical wards undergo at least one advanced imaging test, such as computed tomography (CT), magnetic resonance imaging (MRI), or ultrasound, during their hospitalization [1]. As medical imaging is commonly used and a finite resource within hospitals, inpatients wait hours or sometimes days for a test [2]. Delayed access to medical imaging and imaging-assisted procedures could lead to morbidity and delayed diagnosis. Furthermore, there is some evidence that delays in imaging procedures prolong length-of-stay in hospital, while early imaging procedures can improve outcomes and reduce length-of-stay [2–4].

Both patient and physician-level factors drive decisions to order imaging tests or procedures, and impact delays to their completion. For example, patients with more clinical complexity undergo more tests on average [2]. In selecting an imaging modality, physicians may prioritize easier accessibility and shorter expected wait time, even if alternative test types offer more information [5]. Once ordered, requests are triaged by the radiology department using traditional categorization chosen by the ordering provider (e.g. routine, urgent, stat) [6], but may also consider other factors, such as where the test was ordered. In addition, imaging wait times may also be contingent on hospital factors, such as staff, equipment, and funding. For example, reduced staffing on weekends and in off-hours may result in delayed access to care, longer length-of-stay, and increased morbidity and mortality across a range of conditions–this is one possible explanation for the “weekend effect” [7–11]. Although Ontario has benchmarks for time to test for outpatients (ranging from 2–28 days), there are no such guidelines for inpatient imaging [12].

Imaging procedures are key to timely diagnosis and management of medical inpatients, and delays may result in adverse patient outcomes such as delayed diagnosis and prolonged length-of-stay. However, the degree to which specific factors contribute to imaging delays is not well-understood. In this study, we sought to identify specific patient, physician and system-level factors that contribute to delays in common advanced imaging procedures, and to quantify their contributions to the overall magnitude of delay. A secondary objective was to determine whether imaging delays were associated with longer length of hospital stay for medical inpatients.

## Methods

### Setting, study design, and data sources

This was a retrospective cohort study using health administrative and electronic clinical data from 5 large hospitals in Toronto, Ontario, Canada: St Michael’s Hospital, Mount Sinai Hospital, Toronto General Hospital, Toronto Western Hospital, and Sunnybrook Health Sciences Centre. Of note, the GEMINI database also captures information from two community-based teaching hospitals. However, these hospitals employed a paper-based ordering system during the study period, precluding calculation of time-to-test; these two sites were thus excluded from the study. Patients seen at the included hospitals reflect the diverse population in Toronto, which is generally similar to other medical inpatient populations in Canada, the United States, and Europe [13–15]. The GEMINI database [1] collects patient-level clinical data from individual hospitals and administrative data prepared for the Canadian Institute for Health Information Discharge Abstract Database (CIHI-DAD) [16]. This data includes demographics, diagnoses, interventions, discharge destination, prior research utilization, as well as laboratory and radiology tests and procedures. The “Most responsible physician” (MRP) was the physician most “responsible for the care and treatment of the patient for the majority of the visit to the health care facility”, a definition used by CIHI [16]. Physician characteristics were collected from the publicly available College of Physicians and Surgeons of Ontario (CPSO) physician information database [17]. Research Ethics Board approval was obtained from St. Michael’s Hospital on behalf of all participating hospitals, with a waiver of patient consent for this retrospective study using routinely collected health data.

### Participants

We included all General Internal Medicine (GIM)-admitted patients who had at least one imaging procedure (CT, MRI, ultrasound, or PICC) performed during their hospitalization, and who were discharged between April 1, 2010 and December 31, 2019. In order to evaluate the effect of physician-level characteristics on time-to-test (in multivariable models), we restricted the cohort to hospitalizations with MRPs who had at least 10 hospitalizations in each fiscal year, and for whom information on physician characteristics, such as years in practice or designation as a subspecialist, was available (S1 Fig) [17].

### Outcome measures

The primary outcome was time-to-test, measured as the time elapsed between test ordering and acquisition (CT, MRI, ultrasound) or procedure time (PICC), reported in hours.

We also examined the association of time-to-test with acute length-of-stay after test ordering, in hours. Acute length-of-stay was defined as the difference between total hospital length-of-stay and the count of alternate level of care (ALC) days, which are days spent waiting for a discharge destination [18]. Because delays in imaging tests could not cause the component of length-of-stay that had already occurred prior to test ordering, we further subtracted the time from admission to test ordering to obtain acute length-of-stay after test ordering. Outcomes were measured separately for each test.

### Covariates

We included variables that could impact the time-to-test, at the level of test, hospitalization, patient, and physician. These included system-level variables, measured at test-level: time of ordering relative to admission, whether ordered in the emergency department (ED) or in an Intensive Care Unit (ICU) setting, or ordered during off-hours (evenings or weekends), GIM inpatient census on the day the test was ordered, and designation as bedspaced [19] (patient located on an inpatient unit different from admitting service, excluding ICU). Hospitalization-level variables included age, most responsible diagnosis, Charlson comorbidity index, laboratory-based acute physiology score (LAPS, a score based on presenting laboratory test results that is a validated predictor of mortality when combined with age, comorbidity and sex), neighbourhood income quintile, and fiscal year of hospitalization [20–23]. Each hospitalization was categorized into a clinical condition based on the patient’s principal discharge diagnosis using the Clinical Classifications Software tool, which aggregates ICD-10 diagnoses into 285 mutually-exclusive clinically relevant categories [24]. The analysis included the top 10 categories, and the remaining categories were grouped as “other”. Patient sex was assigned at patient-level. Physician-level variables included sex, years in practice, and designation as a subspecialist as well as each physician’s annual GIM inpatient volume [17]. Hospital site was also included.

### Statistical analysis

Baseline participant characteristics were presented as medians and proportions. We described the volume of tests ordered overall and by test type, count of tests per patient, time to test completion, and time from admission to test ordering. Kolmogorov-Smirnov tests of normality were performed for the primary and secondary outcomes, and confirmed non-normality (p<0.05); for this reason, medians and interquartile ranges were reported.

For each of the four test types (CT, MRI, ultrasound, or PICC), we used separate negative binomial mixed-effect models with random intercepts at the admission, patient, and physician levels (with crossed random intercepts between patients and physicians) to identify predictors of time to test, and to test the hypothesis that imaging delay is associated with prolonged acute length-of-stay after test ordering. Hours spent in hospital waiting for test was the only test-level variable included in models for acute length-of-stay after test ordering.

We reported the association between each variable and the outcome measures as adjusted difference in hours, or average marginal effects, obtained using the “margins” package in R [25]. Average marginal effects are calculated as the mean of partial derivatives of the regression model with respect to each variable and each observation in the data- in contrast with marginal effects at the mean, average marginal effects have the advantage of keeping all other variables at their naturally observed values [26]. For all multilevel models, we measured the degree to which physician-level and patient-level variables contributed to variability in the outcome using the intraclass correlation coefficient (ICC). All hypothesis testing was two-tailed at a p<0.05 significance threshold. All statistical analyses were performed using R version 4.0.2.

## Results

Of the 73,107 hospitalizations included in the final cohort (S1 Fig), about half (47.1%) were women and the median age was 70 years old (Table 1). Of the 129,422 imaging procedures ordered during the study period, 51.7% were CT, 31.0% were ultrasound, 10.6% were MRI, and 6.6% were PICC. Over one-third (39.1%) of patient hospitalizations had at least two imaging procedures, and less than 10% had four or more tests. Almost half (45.9%) of all tests were ordered on the first day of hospitalization: median time-to-order was 38 hours for CT (IQR 7–137), 19 hours for MRI (IQR 5–94), and 14 hours for ultrasound (IQR 2–91); PICC, in contrast, was usually ordered several days into the hospitalization (median 130 hours, IQR 58–276). Approximately one-quarter (26.7%) of tests were ordered for patients in the ED, 7.1% were ordered for patients in the ICU, 35.1% of tests were ordered at night, 22.9% were ordered on the weekend; 22.6% of tests were ordered for patients who were bedspaced.

**Table 1:** Baseline characteristics of patient hospitalizations with at least one advanced imaging test ordered after admission, by type of advanced imaging test.

Characteristic	Total Hospitalizations, n (%) Total N = 73107	Hospitalizations with at least one CT, n (%) Total N = 44403	Hospitalizations with at least one MRI, n (%) Total N = 11492	Hospitalizations with at least one Ultrasound, n (%) Total N = 33254	Hospitalizations with at least one PICC, n (%) Total N = 7602
**Sex, male**	38699 (52.9)	23529 (53.0)	6001 (52.2)	18078 (54.4)	4142 (54.5)
**Age, years**					
18–39	6698 (9.2)	3482 (7.8)	1103 (9.6)	3296 (9.9)	937 (12.3)
40–64	22297 (30.5)	12821 (28.9)	3889 (33.8)	10720 (32.2)	2852 (37.5)
65–79	21724 (29.7)	13576 (30.6)	3834 (33.4)	9616 (28.9)	2112 (27.8)
80 or older	22388 (30.6)	14524 (32.7)	2666 (23.2)	9622 (28.9)	1701 (22.4)
**Charlson Comorbidity Index score**					
0	24406 (33.4)	13470 (30.3)	4833 (42.1)	10343 (31.1)	2373 (31.2)
1	10824 (14.8)	6216 (14.0)	1629 (14.2)	4912 (14.8)	1249 (16.4)
2	14496 (19.8)	9260 (20.9)	1913 (16.6)	6545 (19.7)	1613 (21.2)
3	7279 (10.0)	4403 (9.9)	732 (6.4)	3857 (11.6)	825 (10.9)
≥ 4	16102 (22.0)	11054 (24.9)	2385 (20.8)	7597 (22.8)	1542 (20.3)
**Laboratory-based acute physiology score, median (IQR)**	15.0 (6.0, 28.0)	15.0 (6.0, 28.0)	9.0 (3.0, 19.0)	18.0 (7.0, 32.0)	20.0 (7.0, 33.0)
**Most responsible diagnosis**					
Pneumonia	3361 (4.6)	2575 (5.8)	144 (1.3)	1251 (3.8)	188 (2.5)
Congestive heart failure	3267 (4.5)	1758 (4.0)	75 (0.7)	2110 (6.3)	174 (2.3)
Urinary tract infection	3125 (4.3)	1324 (3.0)	147 (1.3)	2010 (6.0)	404 (5.3)
Cerebral infarction	2647 (3.6)	1645 (3.7)	1576 (13.7)	438 (1.3)	59 (0.8)
Chronic obstructive pulmonary disease	2560 (3.5)	1927 (4.3)	68 (0.6)	965 (2.9)	122 (1.6)
Sepsis	2071 (2.8)	1313 (3.0)	157 (1.4)	1141 (3.4)	608 (8.0)
Neurocognitive disorders	2023 (2.8)	1349 (3.0)	492 (4.3)	749 (2.3)	69 (0.9)
Secondary malignancies	1891 (2.6)	1511 (3.4)	529 (4.6)	533 (1.6)	112 (1.5)
Acute and unspecified renal failure	1753 (2.4)	631 (1.4)	61 (0.5)	1452 (4.4)	134 (1.8)
Gastrointestinal bleed	1528 (2.1)	965 (2.2)	52 (0.5)	723 (2.2)	110 (1.4)
**Acute length-of-stay, days, median (IQR)**	9.3 (4.8, 18.7)	9.7 (5.2, 19.6)	8.4 (4.4, 16.8)	8.0 (4.3, 16.1)	14.4 (7.8, 28.5)
Acute length-of-stay after test ordering*, days, median (IQR)	5.4 (2.9, 10.1)	5.7 (3.0, 10.6)	5.0 (2.6, 9.2)	5.2 (2.8, 9.5)	5.8 (2.9, 11.4)
**Neighbourhood income quintile**					
Q1	16048 (22.0)	9710 (21.9)	2393 (20.8)	7599 (22.9)	1594 (21.0)
Q2	12044 (16.5)	7491 (16.9)	1693 (14.7)	5417 (16.3)	1276 (16.8)
Q3	11354 (15.5)	6940 (15.6)	1733 (15.1)	5177 (15.6)	1175 (15.5)
Q4	10691 (14.6)	6554 (14.8)	1622 (14.1)	4798 (14.4)	1201 (15.8)
Q5	12693 (17.4)	7829 (17.6)	1996 (17.4)	5485 (16.5)	1292 (17.0)
Information not available	10277 (14.1)	5879 (13.2)	2055 (17.9)	4778 (14.4)	1064 (14.0)
**Advanced imaging tests/procedures during hospitalization**					
One	44541 (60.9)	20413 (46.0)	4918 (42.8)	16905 (50.8)	2305 (30.3)
Two	16349 (22.4)	12640 (28.5)	2737 (23.8)	8339 (25.1)	1886 (24.8)
Three	6391 (8.7)	5729 (12.9)	1614 (14.0)	3771 (11.3)	1139 (15.0)
Four or more	5826 (8.0)	5621 (12.7)	2223 (19.3)	4239 (12.7)	2272 (29.9)
**Frequency of test type during hospitalization**					
At least one CT	44403 (60.7)	44403 (100.0)	4937 (43.0)	12553 (37.7)	3855 (50.7)
At least one MRI	11492 (15.7)	4937 (11.1)	11492 (100.0)	2608 (7.8)	955 (12.6)
At least one ultrasound	33254 (45.5)	12553 (28.3)	2608 (22.7)	33254 (100.0)	3180 (41.8)
At least one PICC	7602 (10.4)	3855 (8.7)	955 (8.3)	3180 (9.6)	7602 (100.0)

CT–computed tomography, MRI–magnetic resonance imaging, PICC–peripherally-inserted central catheter. *Q1 is lowest income and Q5 is highest income. IQR–interquartile range.

*Defined as difference between acute length-of-stay and time to test ordering (in days), excluded N = 530 hospitalizations.

### Time-to-test

Unadjusted time-to-test was shortest for CT (median 7 hours, IQR 2–22) and longest for MRI (22 hours, IQR 10–46) and PICC (22 hours, IQR 5–46). Test-level factors accounted for most of the variability in time-to-test for CT (76.1%), ultrasound (90.7%), MRI (78.8%), and PICC (95.5%) (data not in table). Patient factors (measured at patient or hospitalization-levels) accounted for some of the variability in time to CT (19.9%), ultrasound (7.6%), MRI (16.0%), and PICC (2.0%) (data not in table). Measured physician factors contributed little to the time to CT (4.0%), ultrasound (1.7%), MRI (5.2%), and PICC (2.5%) (data not in table). Physician-level variables also did not show a consistent association with test delays (Table 2).

**Table 2:** Adjusted difference in time-to-test (95% confidence interval), in hours.

Variable	CT	MRI	Ultrasound	PICC
**Time to test order**				
<1 day	Ref.	Ref.	Ref.	Ref.
1 to <2 days	**1.7 (1.0, 2.3)**	**3.9 (1.9, 5.9)**	**3.3 (2.2, 4.5)**	1.1 (-2.7, 4.9)
2 to <4 days	**1.5 (0.9, 2.1)**	**5.2 (3.1, 7.3)**	**4.5 (3.4, 5.7)**	1.7 (-1.7, 5.2)
≥ 4 days	**1.0 (0.5, 1.5)**	**12.8 (10.8, 14.8)**	**5.5 (4.5, 6.5)**	**4.3 (1.1, 7.4)**
**Location test ordered** ^**†**^				
Emergency department	**-6.7 (-7.2, -6.3)**	**-6.2 (-7.8, -4.5)**	**-7.5 (-8.3, -6.7)**	**-7.3 (-11.2, -3.5)**
Intensive care unit	**-7.3 (-7.8, -6.8)**	-2.5 (-5.5, 0.5)	**-10.4 (-11.3, -9.5)**	**3.9 (0.3, 7.5)**
Bedspaced	**-1.2 (-1.6, -0.7)**	**-5.0 (-6.5, -3.5)**	**-1.8 (-2.7, -1.0)**	-1.5 (-3.7, 0.7)
**GIM census on day ordered**	**-0.1 (-0.1, -0.1)**	**-0.1 (-0.1, 0.0)**	**-0.1 (-0.1, -0.1)**	**-0.3 (-0.3, -0.2)**
Ordered on weekend*	**8.2 (7.7, 8.8)**	**8.9 (7.2, 10.5)**	**20.9 (19.9, 22.0)**	**21.4 (17.5, 25.3)**
Ordered at night**	0.2 (-0.2, 0.6)	**1.6 (0.2, 2.9)**	**3.5 (2.8, 4.2)**	2.2 (-0.2, 4.6)
Hospital***				
Hospital 1	Ref.	Ref.	Ref.	Ref.
Hospital 2	**10.5 (9.6, 11.4)**	**-**	**-8.1 (-9.8, -6.4)**	**12.2 (7.4, 17.0)**
Hospital 3	**12.2 (11.5, 12.8)**	**-13.9 (-15.5, -12.4)**	**-9.5 (-10.6, -8.4)**	**14.3 (10.9, 17.5)**
Hospital 4	**4.5 (4.0, 4.9)**	**3.1 (0.9, 5.3)**	**-6.6 (-7.9, -5.3)**	**4.4 (1.5, 7.2)**
Hospital 5	**6.2 (5.7, 6.7)**	**7**.**9 (5.5, 10.4)**	**-7.9 (-9.2, -6.6)**	-2.1 (-4.7, 0.4)
**Patient age**				
≤ 39 years old	Ref.	Ref.	Ref.	Ref.
40–64 years old	**1.8 (1.1, 2.4)**	0.5 (-1.3, 2.4)	**1.9 (0.9, 2.9)**	1.3 (-1.5, 4.1)
65–79 years old	**2.4 (1.8, 3.1)**	**5.7 (3.7, 7.7)**	**3.2 (2.1, 4.2)**	1.3 (-1.7, 4.3)
≥ 80 years old	**2.5 (1.8, 3.1)**	**8.3 (6.1, 10.5)**	**3.4 (2.3, 4.5)**	2.7 (-0.5, 5.9)
**Charlson comorbidity score**				
0	Ref.	Ref.	Ref.	Ref.
1	0.1 (-0.4, 0.6)	**2.1 (0.3, 3.8)**	0.5 (-0.4, 1.5)	0.1 (-2.6, 2.8)
2	**2.3 (1.8, 2.8)**	**2.8 (1.1, 4.5)**	0.9 (0.0, 1.8)	0.5 (-2.1, 3.0)
3	**1.6 (0.9, 2.3)**	2.5 (-0.1, 5.1)	**1.6 (0.5, 2.7)**	0.3 (-3.0, 3.5)
≥ 4	**3.4 (2.9, 3.9)**	**5.7 (3.8, 7.7)**	**1.8 (0.9, 2.7)**	-0.1 (-2.8, 2.6)
**LAPS**	0.0 (0.0, 0.0)	0.1 (0.0, 0.1)	0.0 (0.0, 0.0)	0.0 (-0.1, 0.0)
**Most responsible diagnosis** ^**‡**^				
Pneumonia	**-1.4 (-2.1, -0.7)**	2.1 (-3.7, 7.9)	**-1.7 (-3.2, -0.3)**	1.5 (-4.5, 7.6)
Congestive heart failure	**-2.4 (-3.2, -1.5)**	2.1 (-6.2, 10.4)	0.3 (-1.1, 1.6)	-2.9 (-8.4, 2.6)
Urinary tract infection	**-3.0 (-3.9, -2.1)**	-1.4 (-6.4, 3.6)	**-1.8 (-3.0, -0.6)**	**-4.1 (-7.6, -0.7)**
Cerebral infarction	**-3.2 (-3.9, -2.4)**	**-2.0 (-3.7, -0.3)**	**4.2 (1.2, 7.3)**	**-7.8 (-15.2, -0.4)**
COPD	**-1.5 (-2.4, -0.6)**	-5.0 (-12.0, 2.1)	0.5 (-1.3, 2.4)	-0.6 (-8.0, 6.7)
Sepsis	**-2.7 (-3.6, -1.9)**	**7.2 (1.2, 13.3)**	-0.6 (-2.4, 0.9)	2.4 (-1.0, 5.8)
Neurocognitive disorders	**-1.3 (-2.3, -0.4)**	**6.8 (3.3, 10.4)**	**8.9 (6.1, 11.6)**	3.1 (-6.3, 12.5)
Secondary malignancies	**3.0 (1.9, 4.1)**	**-8.2 (-10.8, -5.6)**	0.8 (-1.9, 3.4)	2.0 (-6.9, 10.9)
Acute renal failure	**-2.0 (-3.4, -0.7)**	-2.1 (-9.6, 5.5)	**-1.7 (-3.1, -0.3)**	**-10.3 (-14.8, -0.58)**
Gastrointestinal bleed	1.3 (0.0, 2.6)	3.9 (-6.1, 13.9)	**2.9 (0.6, 5.1)**	-3.9 (-10.5, 2.7)
**Neighbourhood income quintile**				
Q1	Ref.	Ref.	Ref.	Ref.
Q2	-0.1 (-0.7, 0.5)	**-3.4 (-5.4, -1.3)**	-0.6 (-1.6, 0.3)	-1.3 (-4.3, 1.6)
Q3	-0.4 (-1.0, 0.2)	**-4.1 (-6.1, -2.2)**	**-1.4 (-2.3, -0.4)**	-0.5 (-3.6, 2.6)
Q4	-0.6 (-1.2, 0.0)	-2.1 (-4.1, 0.0)	**-1.5 (-2.5, -0.5)**	**-4.2 (-7.0, -1.3)**
Q5	-0.5 (-1.1, 0.0)	**-5.8 (-7.7, -4.0)**	**-1.1 (-2.1, -0.2)**	-0.5 (-3.5, 2.5)
Information not available	**-0.9 (-1.5, -0.2)**	**2.6 (0.4, 4.9)**	**-1.7 (-2.8, -0.6)**	**-6.8 (-9.9, -3.8)**
**Patient sex–male** ^**‡‡**^	0.2 (-0.1, 0.6)	**1.3 (0.1, 2.5)**	**0.7 (0.1, 1.3)**	**1.9 (0.1, 3.6)**
**Physician sex–male** ^**‡‡**^	0.0 (-0.4, 0.4)	0.1 (-1.2, 1.5)	-0.6 (-1.3, 0.0)	0.7 (-1.3, 2.7)
**Subspecialist** ^**††**^	-0.2 (-0.6, 0.3)	-0.1 (-1.8, 1.7)	0.9 (0.0, 1.8)	-1.1 (-3.4, 1.2)
**Years in practice**				
0–4 years	Ref.	Ref.	Ref.	Ref.
5–14 years	**-0.5 (-1.0, -0.1)**	-0.1 (-1.8, 1.6)	-0.5 (-1.3, 0.3)	2.2 (-0.3, 4.6)
15–24 years	0.2 (-0.3, 0.7)	-1.1 (-2.8, 0.7)	-0.1 (-1.0, 0.8)	1.8 (-0.8, 4.4)
≥ 25 years	**0.7 (0.1, 1.3)**	0.3 (-2.0, 2.5)	**1.4 (0.3, 2.5)**	1.1 (-1.9, 4.1)
**Annual patient volume**				
Q1	Ref.	Ref.	Ref.	Ref.
Q2	0.6 (-0.1, 1.4)	0.9 (-1.7, 3.5)	1.2 (0.0, 2.5)	-0.1 (-3.8, 3.6)
Q3	0.4 (-0.3, 1.1)	**2.9 (0.4, 5.4)**	1.0 (-0.2, 2.2)	0.1 (-3.5, 3.7)
Q4	0.5 (-0.2, 1.2)	1.9 (-0.5, 4.3)	**1.3 (0.2, 2.5)**	(-3.3, 3.7)

Bolding indicates statistically significant differences. Negative values indicate characteristic associated with a shorter time-to-test. In addition to all listed variables, time-to-test was adjusted for hospital and fiscal year of hospitalization (not shown). CT–computed tomography; MRI–magnetic resonance imaging; PICC–peripherally-inserted central catheter; GIM–general internal medicine; COPD–chronic obstructive pulmonary disease. LAPS–laboratory-based acute physiology score.

^**†**^Referents were not ordered in Emergency Department, not ordered in Intensive Care Unit, and not ordered while bedspaced.

*Referent was weekday.

**Referent was day. Q1 is lowest income and Q5 is highest income.

*** Hospital 2 is missing for MRI because this hospital uses a paper-based ordering system for MRI.

^**‡**^For all admitting diagnoses, referent was absence of that diagnosis.

^**‡‡**^Referent was female.

^**††**^Referent was non-specialist.

### Test-level characteristics and hospital sites

**Fig 1:**
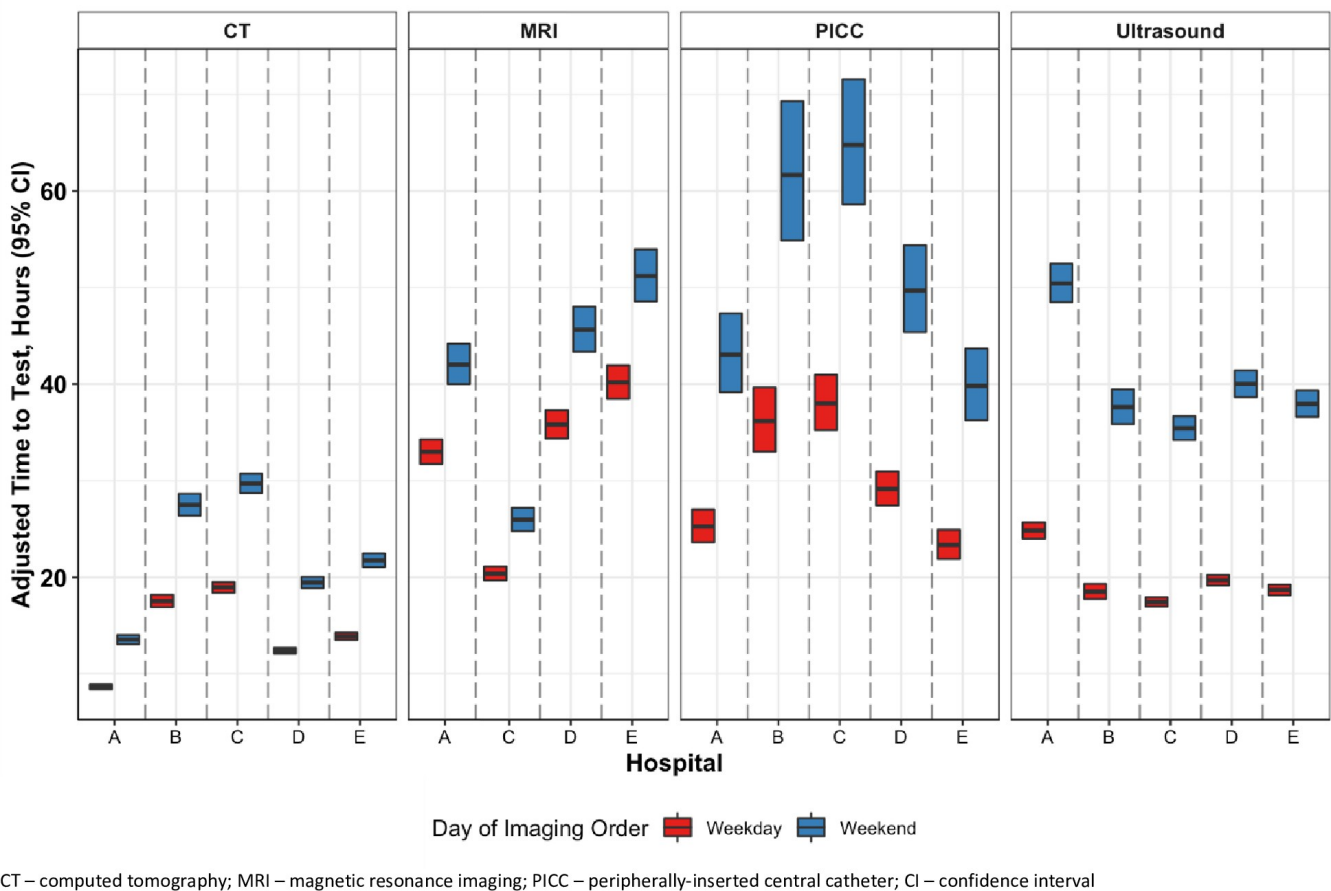
Hospital-specific adjusted time-to-test (median, hours, +/- 95% confidence intervals) for imaging procedures for patients admitted to General Internal Medicine. Note: Hospital B did not contribute information on time to MRI as it uses a paper-based ordering system.

The variables accounting for the greatest delay in time-to-test related to where and when the test was ordered. Notably, adjusted time-to-test showed large differences across hospital sites: 12.2 hours for CT, 21.8 hours for MRI, 9.5 hours for ultrasound and 14.3 hours for PICC (Table 2). Tests ordered on weekends also took considerably longer: an additional 8.2 hours for CT, 8.9 hours for MRI, 20.9 hours for ultrasound and 21.4 hours for PICC. Each hospital’s adjusted time-to-test, broken down by weekend or weekday ordering (Fig 1), confirms that the additional weekend-related delay occurred at all sites and for all test types, and was typically largest for ultrasound and PICC.

Patients whose imaging was ordered from a ward location waited considerably longer than if it was ordered from the emergency department or an ICU location. This difference was largest for ultrasounds, which happened 7.5 hours faster if ordered in the emergency department, and 10.4 hours faster if ordered from an ICU. Additionally, ordering tests later in the hospitalization was associated with prolonged time-to-test for all test types, with additional delays of 12.8 hours for MRI, 5.5 hours for ultrasound and 4.3 hours for PICC if a test was ordered on day 4 or later.

Between 2010 and 2019, time-to-test decreased significantly for PICC (adjusted difference -9.3 hours, 95% CI -14.5 to -4.1), increased for CT (adjusted difference 2.8 hours, 95% CI 1.9–3.8), and did not change significantly for MRI or ultrasound.

### Patient characteristics

After adjusting for all covariates, older patients, especially those aged 80 years and older, had significantly longer time-to-test for CT (2.5 hours), MRI (8.3 hours), and ultrasound (3.4 hours). A higher count of comorbidities was also associated with a longer time-to-test; those with a Charlson score of ≥ 4 waited an additional 5.7 hours for MRI and 3.4 hours for CT. Those residing in the lowest income neighborhoods had a longer time-to-test than those in the highest income neighborhoods, for MRI (5.8 hours) and ultrasound (1.1 hours). Male patient sex was associated with a small delay for PICC line insertion (1.9 hours), MRI (1.3 hours), and ultrasound (0.7 hours).

### Acute length-of-stay after test ordering

The median acute length-of-stay after test ordering was 5.4 days (IQR 2.9–10.1) overall; this was 5.2 days (IQR 2.8–9.5 days) for ultrasound, 5.0 days (IQR 2.6–9.2) for MRI, 5.7 days (IQR 3.0–10.6) days for CT, and 5.8 days (IQR 2.9–11.4) for PICC. After adjusting for all other variables, each additional hour spent waiting for a test was associated with an increased length-of-stay after test ordering of 1.2 hours for MRI (95% CI 1.0–1.3), 1.1 hours for PICC (95% CI 0.92–1.4), 0.8 hours for ultrasound (95% CI 0.7–0.9), and 0.4 hours for CT (95% CI 0.3–0.5).

## Discussion

In our study of 73,107 general medical hospitalizations across 5 hospitals, the greatest contributors to testing delays were hospital site and test-level factors such as ordering time and unit within the hospital. Specifically, hospital site (up to 22 additional hours), unit within the hospital (up to 10 additional hours), the timing of test ordering relative to admission (up to 13 additional hours), and ordering during weekends (up to 21 additional hours) contributed the most to variability in time-to-test. Older patient age, greater comorbidity and residence in a low-income neighborhood were associated with longer time-to-test for some test types, even after adjusting for most responsible diagnosis and severity of illness. Physician characteristics were minimally associated with testing delay.

Although there have been a few studies of the association between imaging delays and hospital length of stay, we did not find any previous literature on contributors to imaging delays for medical inpatients. In our study, we quantified the impact of patient factors such as clinical complexity, and physician and system factors, on time-to-test. We found that some of the most important factors determining time-to-test for inpatients were the hospital, time, and within-hospital patient location at the time of test ordering–in short, characteristics that relate to hospital testing capacity and workflows for test prioritization. For example, the number of MRI machines within the hospital, the robustness of the sonographer workforce, and the time allocated to inpatient versus outpatient testing may each impact a hospital’s capacity for timely imaging. Our study findings also provide context to understand the well-described “weekend effect”, in which a higher mortality is observed for patients admitted on weekends than those admitted on weekdays [8,10,11]. We found that ordering tests on the weekend was associated with an increased time-to-test of up to 21.4 hours; delayed radiological diagnosis or intervention could contribute to the poor outcomes of weekend-admitted patients. Our findings have highlighted an opportunity to improve access to radiology tests and procedures through system-level changes.

A few single-site observational and decision modelling studies have suggested that length-of-stay for medical inpatients is prolonged by advanced imaging, even after adjustment for comorbidity, illness severity, and risk of death [2,4,5]. We found that each additional hour spent waiting for an MRI was associated with a 1.2-hour increase in length-of-stay, which is similar to 1.12 days (for each day spent waiting) reported in a single-hospital study in Ireland [5]. CT has been shown to have shorter delays than MRI or ultrasound, which is a finding replicated in our study, and may reflect easier access to CT during off-hours [2]. Indeed, additional delays related to test or hospitalization characteristics were often greatest for test types that typically take longer to obtain (MRI, ultrasound, PICC), suggesting the potential for greater inequity or care differences when using less-accessible imaging modalities.

Early advanced imaging has been associated with reduced length-of-stay, as well as improved patient outcomes [3–5,9,27]. Weekend-related imaging delays, as measured and presented in this study, will be incorporated into a province-wide general internal medicine performance dashboard [28]. Hospital leaders may further consider how delays in imaging at their institution compare to their peer hospitals, and the effect this may have on their patients’ relative and absolute length-of-stay. Hospital initiatives to improve access to imaging procedures–for instance, through increased radiology coverage on weekends [9]–could shorten length-of-stay and improve other patient outcomes.

Further research is needed to understand what may be driving delays at the patient-level. We found that older patients and those with more medical complexity tended to wait longer for tests, even after adjusting for severity of illness, comorbidity count and the most common diagnoses. It is possible that longer wait times reflect less urgent indications in older or more chronically ill patients. Alternatively, this could reflect biases in how patients are prioritized for a test. Our finding that patients from high-income neighborhood had shorter waiting times for some tests also raises equity concerns. Biases could affect ordering physician advocacy, and thus test prioritization. The ordering physician selects the urgency of the test and provides the indication. In addition to how the original imaging request is worded, biases may affect whether and how often ordering physicians contact the imaging department or radiologist on-call to request a patient’s test be further expedited. Furthermore, patients serve as key advocates for themselves, and certain groups may be less capable or effective in doing so. Beyond physician decision-making, patient factors that could contribute indirectly to delays include patients leaving their home unit unpredictably, agitation in the radiology suite, an inability to provide consent, or a sudden refusal to proceed with a test to which they had previously consented.

### Strengths and limitations

Strengths of our study include its large sample size across 5 hospitals and 10 years, using clinical and administrative data source with excellent overall reliability [29]. There are several limitations to our study. First, the indication and urgency for imaging tests ordered were unavailable, and impact both time-to-test and length-of-stay in hospital. To address this, we accounted for illness severity (via the LAPS) and the most common diagnoses. In future studies, incorporating the ordering indications and Canadian Triage and Acuity Scale level, as well as conducting chart reviews, would help clarify the urgency of a given test order and gauge to what extent prolongations in length-of-stay can be directly attributed to imaging delays. Second, our data sources only captured completed tests, hence our estimates of testing delays do not account for test cancellation and re-ordering, which further prolongs the time to some tests. Third, while we could identify the most responsible physician, resident physicians often order investigations, especially during off-hours. Physician misattribution could lead to underestimation of the physician-level effect on all outcomes. Fourth, our results do not account for any additional delays between the time of test completion and an available test report. Imaging report turnaround time can be significant–up to 26 hours for MRI or ultrasound at one hospital in Seattle–and can also prolong length-of-stay [30]. Finally, the specific predictors identified, and the magnitude of their contributions may not be generalizable outside our system, particularly where hospital funding models and care processes differ. Yet, our approach can be applied by all hospital systems to inform quality improvement and reduce imaging-related delays locally.

## Conclusions

Overall, we identified key factors that affect time-to-test for imaging procedures for medical inpatients. Patient factors, such as increasing age and greater comorbid illness, were associated with increased delays, while physician factors contributed minimally to wait times. The greatest contributors to time-to-test were system factors related to where a test was ordered (hospital site and unit) and when it was ordered (weekend versus weekday, and timing relative to admission). Given that wait times contribute to prolonged length-of-stay and quality of care, hospital leaders should consider applying our novel methods to identify imaging-related delays in their system, and use this as a springboard for quality improvement.

## Supporting information

S1 Fig Creation of the study cohort and cohort used in the multivariable model. (DOCX)
