# Supplementary material for: Imaging delays among medical inpatients in Toronto, Ontario: A cohort study
Source: PLoS One. 2023 Feb 3;18(2):e0281327. doi: 10.1371/journal.pone.0281327 (PMC9897551; doi:10.1371/journal.pone.0281327)
Supplement: S1 Fig — (DOCX) [file pone.0281327.s001.docx]

**Supplemental Figure 1. Creation of the study cohort and cohort used in the multivariable model.**

196,819 hospitalizations on General Internal Medicine between April 1, 2010 and December 31, 2019

No tests ordered during hospitalization (n = 22,999 hospitalizations)

77,562 hospitalizations (56,173 patients, 137,993 tests)
included in the study cohort

Test(s) ordered before hospitalization
(n = 96,258 hospitalizations)

Valid CPSO information unavailable (n = 4,126 hospitalizations)

MRP with fewer than 10 hospitalizations per year (n = 329 hospitalizations)

73,107 hospitalizations (52,850 patients, 129,422 tests)
included for time-to-test analysis

Excluded as test potentially ordered during alternate level of care (ALC) period (Acute LOS- time to test ordering <0 day). (n = 530 hospitalizations)

72,577 hospitalizations (52,481 patients, 125,999 tests)
included for acute LOS after test ordering analysis

MRP – most responsible provider, CPSO – College of Physicians and Surgeons of Ontario
